# Uncovering the ferroptosis related mechanism of laduviglusib in the cell-type-specific targets of the striatum in Huntington’s disease

**DOI:** 10.1186/s12864-024-10534-5

**Published:** 2024-06-25

**Authors:** Mei Liu, Jinlan Zhao, Chengcheng Xue, Jun Yang, Li Ying

**Affiliations:** 1 Jiangxi Key Laboratory of Neurological Diseases, Department of Neurosurgery, The First Affiliated Hospital, Jiangxi Medical College, Nanchang University, Nanchang, Jiangxi China; 2 Joint Laboratory for Translational Cancer Research of Chinese Medicine of the Ministry of Education of the People’s Republic of China, School of Pharmaceutical Science, International Institute for Translational Chinese Medicine, Guangzhou University of Chinese Medicine, Guangzhou, China; 3 Department of Neurology, First Affiliated Hospital of Nanchang University, Nanchang, China

**Keywords:** Huntington’s disease, Laduviglusib, Ferroptosis, Molecular docking, Single-nuclei RNA sequencing

## Abstract

Huntington’s disease (HD) is a dominantly inherited neurodegenerative disorder featured by abnormal movements, arising from the extensive neuronal loss and glial dysfunction in the striatum. Although the causes and pathogenetic mechanisms of HD are well established, the development of disease-modifying pharmacological therapies for HD remains a formidable challenge. Laduviglusib has demonstrated neuroprotective effects through the enhancement of mitochondrial function in the striatum of HD animal models. Ferroptosis is a nonapoptotic form of cell death that occurs as a consequence of lethal iron-dependent lipid peroxidation and mitochondrial dysfunction. However, the ferroptosis-related mechanisms underlying the neuroprotective effects of laduviglusib in the striatum of HD patients remain largely uncharted. In this study, we leveraged single-nucleus RNA sequencing data obtained from the striatum of HD patients in stages 2–4 to identify differentially expressed genes within distinct cell-type. We subsequently integrated these differentially expressed genes of HD, laduviglusib target genes and ferroptosis-related genes to predict the ferroptosis-related mechanisms underpinning the neuroprotective effects of laduviglusib in HD patients. The Kyoto Encyclopedia of Genes and Genomes (KEGG) and Gene Ontology (GO) analyses unveiled that the effects of laduviglusib on direct pathway striatal projection neurons (dSPNs) is mainly associated with Th17 cell differentiation pathways. Conversely, its impact on indirect pathway striatal projection neurons (iSPNs) extends to the Neurotrophin signaling pathway, FoxO signaling pathway, and reactive oxygen species pathway. In microglia, laduviglusib appears to contribute to HD pathology via mechanisms related to Th17 cell differentiation and the FoxO signaling pathway. Further, molecular docking results indicated favorable binding of laduviglusib with PARP1 (associated with dSPNs and iSPNs), SCD (associated with astrocytes), ALOX5 (associated with microglia), and HIF1A (associated with dSPNs, iSPNs, and microglia). In addition, the KEGG results suggest that laduviglusib may enhance mitochondrial function and protect against neuronal loss by targeting ferroptosis-related signaling pathways, particularly mediated by ALOX5 in microglia. These findings provide valuable insights into the potential mechanisms through which laduviglusib exerts its effects on distinct cell-types within the HD striatum.

**Supplementary Information:**

The online version contains supplementary material available at 10.1186/s12864-024-10534-5.

## Introduction

Huntington’s disease (HD) represents a dominantly inherited neurodegenerative condition featured by the expansion of a CAG short tandem repeat in exon 1 of the huntingtin (HTT) gene. This condition is hallmarked by extensive neuronal loss of striatal spiny projection neurons (SPNs), accompanied by a pronounced inflammatory response involving reactive striatal glia [1, 2]. Depending on the severity of the striatal neuropathological involvement and clinical disability, HD is designated into five grades (0–4) in ascending order of severity [1, 3]. Additionally, post-mortem analyses of HD patient tissue have demonstrated the gradual emergence of profound reactive astrocytes in grades 2–4 [4]. Microglial activation, as observed in post-mortem studies, correlates with disease severity, contributing to ongoing neuronal degeneration in HD [5]. Although the pathogenetic causes of HD are well understood, there is currently no approved disease-modifying treatment for HD [6].

Laduviglusib, also known as CHIR99201, is a highly selective inhibitor of Glycogen synthase kinase 3 (GSK3), a protein implicated in cell survival. Laduviglusib has been shown to enhance insulin glucose transporter activation, pancreatic β cell proliferation, and survival [7, 8]. Beyond its role in GSK3 regulation, laduviglusib also modulates the Wnt signaling pathway by directly inducing β-catenin phosphorylation; Consequently, laduviglusib, in conjunction with other compounds, often acts as a mediator in differentiating stem cells into neurons [9–11]. Furthermore, laduviglusib exhibited potential therapeutic relevance in neurological disorders, such as ischemic stroke, Alzheimer’s disease. In ischemic stroke, the combined use of laduviglusib with other compounds has been shown to promote the transformation of macrophages into neurons both in vitro and in vivo, leading to improved neurological function [12]. In Alzheimer’s disease, laduviglusib has been associated with decreased tau phosphorylation in human glutamatergic neurons, suggesting its capacity to counteract tau pathology, particularly in neurons [13]. Another in vitro study demonstrated that laduviglusib rescued tubulin polymerization in ankyrin 3-repressed cells, a perturbation potentially linked to psychiatric illnesses, by pharmacological inhibiting collapsin response mediator protein 2 (CRMP2) activity [14]. These collectively works corroborate the protective role of laduviglusib in neurological disease by potentiating cell survival. Besides, several studies have claimed that treated with laduviglusib significantly inhibits oxidative damage, including the upregulation of reactive oxygen species (ROS) levels, disruption of mitochondrial membrane potential, excessive lipid peroxidation, and the reduction of superoxide dismutase (SOD) activity [15], indicating the potential involvement of laduviglusib in oxidative-related dysfunction. Several features associated with laduviglusib, such as ROS levels, mitochondrial dysfunction, increased lipid peroxidation, and reduced SOD activity, are closely related to ferroptosis [16].

Ferroptosis is a nonapoptotic form of cell death that occurs as a consequence of lethal iron-dependent lipid peroxidation and mitochondrial dysfunction [16]. A bulk of evidences suggest that ferroptosis is integral to the pathogenesis of HD. For example, previous study described the accumulation of toxic iron in neurons in HD mice compared to wild-type mice, which consequently induces neurodegenerative processes [17]. Another MRI study on HD patients also showed iron accumulation in the brain [18]. Additionally, research by Sonal et al. has elucidated the critical role of iron in regulating mitochondrial metabolism in HD, as evidenced by increased mitochondrial iron content, elevated levels of the iron uptake protein mitoferrin 2, and reduced iron-sulfur cluster synthesis protein frataxin in brain tissue of HD patients and HD mice [19]. Enriched mitochondrial isolates from the brains of HD mice displayed defects in membrane potential, oxygen uptake, and increased lipid peroxidation [19]. Likewise, the accumulation of lipid peroxidation adducts also found in the caudate nucleus and putamen (CPu) region of the HD striatum, whose functional integrity critical associated with abnormal movements in HD [20, 21]. The aforementioned evidences, observed in both HD animal models and human patients, underscore the strong correlation between ferroptosis and biological and clinical features of HD. Furthermore, pharmacological reagents that modulated morphological and biochemical features implicated in ferroptosis exhibit great potential in clinical therapies, especially in HD [22]. Previous studies exploring ferrostatin-1, a selective inhibitor of ferroptosis, have demonstrated its ability to prevent iron-dependent oxidative stress and rescue neuronal cell death in cellular models of HD [23]. This study suggests the efficacy of regulation of ferroptosis in improving HD-related features.

In HD, the neuroprotective effects of laduviglusib benefits are attributed to its influence on the calpastatin (CAST)–calpain–dynamin-related protein 1 (Drp1) signaling axis [24]. Numerous studies have highlighted the interplay between calpain, Drp1, and ferroptosis. For instance, polygalain extract, tenuifolin, has been demonstrated to mitigate ferroptosis by modulating calpain activity, potentially preventing the progression of Alzheimer’s disease-like phenotypes [25]. Activated calpain mediates calcium influx and caspase activity, which has been implicated in radiation-induced endothelial cell ferroptosis [26]. The modulation of calpain activity is implicated in the pathogenesis of systemic sclerosis (SSc)-associated ferroptosis [27]. Consistently, enhanced Drp1 activity facilitates ferroptosis in ischemia/reperfusion injury [28]. It has been reported that Drp1-regulated mitochondrial fission is implicated in ferroptosis [29]. Moreover, in hepatocellular carcinoma (HCC) cells, Drp1 can protect against ferroptosis by modulating mitochondrial function [30]. These findings collectively suggest that the activation of calpain/Drp1 may lead to mitochondrial and lysosomal damage, thereby promoting or exacerbating ferroptosis.

In the present study, we conducted a comprehensive analysis by utilizing single-cell sequencing data obtained from post-mortem samples of HD patients in stages 2–4. Through integration with laduviglusib target genes, our aim was to elucidate the potential cellular targets of laduviglusib within the HD CPu. Subsequently, through enrichment analysis, we explored the signaling pathways affected by laduviglusib in the treatment of HD. Our investigation revealed that laduviglusib exerts regulatory effects on distinct cell-types in HD, particularly in direct pathway striatal projection neurons (dSPNs), indirect pathway striatal projection neurons (iSPNs), astrocytes, and microglia, where it influences processes related to ribose metabolism and oxidative stress response. Building upon previous research, our findings suggest a pivotal role for ferroptosis in these processes. Molecular docking results indicated strong binding affinity of laduviglusib with PARP1 (associated with dSPNs and iSPNs), SCD (associated with astrocytes), AR (associated with astrocytes), ALOX5 (associated with microglia), and HIF1A (associated with dSPNs, iSPNs, astrocytes, and microglia). With the integration of ferroptosis-related data, we postulate that laduviglusib may exert its therapeutic effects in HD by targeting ferroptosis-associated signaling pathways, particularly those mediated by ALOX5 in microglia. This targeting strategy aims to enhance mitochondrial functionality, protect against neuronal loss, and mitigate the neuropathological changes associated with HD. As a result, our study highlights the presence of a potentially druggable pathway involving neuronal and glial ferroptosis in the pathogenesis of HD. Research on this small molecule drug, laduviglusib, might serve as a pioneering step in the development of treatments for HD.

## Methods

### Collection of target genes for different cell-types in HD

We obtained cell-type-specific gene expression profiles from brain single-nucleus RNA sequencing (snRNA-seq) data of Huntington’s disease (HD) patients, as reported by Lee et al. (GSE152058) [1]. Differentially expressed genes were identified by comparing conditions with an absolute log-fold change > 0.1 and a false discovery rate (FDR) < 0.001 against their respective controls for each cell-type. The selection of these parameters was primarily based on the foundational work of Lee and colleagues.

### Collection of laduviglusib target genes

We retrieved the Simplified Molecular Input Line Entry System (SMILES) structure of laduviglusib from the pharmacology database (https://pubchem.ncbi.nlm.nih.gov/). To predict the target genes of laduviglusib, we queried multiple databases, including PharmMapper (http://www.lilab-ecust.cn/pharmmapper/), SEA (https://sea.bkslab.org/), SuperPred (https://prediction.charite.de/), and SwissTargetPrediction (http://swisstargetprediction.ch/), using the SMILES structure of laduviglusib. The final set of laduviglusib target genes was derived from the sum of results across these databases after removing duplicate entries.

### Collection of ferroptosis-related genes

We obtained a collection of ferroptosis-associated genes from the FerrDb V2 database (http://www.zhounan.org/ferrdb/current/) [31]. This collection included human genes that either promote, inhibit, or are associated with the occurrence of ferroptosis.

### Gene ontology (GO) and kyoto encyclopedia of genes and genomes (KEGG) analyses

We performed enrichment analysis of Gene Ontology and KEGG pathways to elucidate the biological processes (BP), cellular components (CC), molecular functions (MF), and pathways associated with the significant genes. The analysis was conducted using the clusterProfiler package (version 4.2.2) and the ggplot2 package (version 3.3.6) in R studio. All the mentioned analyses were carried out using R version 4.1.2.

### Construction of protein-protein interaction (PPI) networks for ferroptosis-related genes in laduviglusib against each cell-type in HD

To explore potential interactions between proteins encoded by the target genes, we constructed a PPI network using the online database STRING (https://cn.string-db.org/). A confidence score cut-off of 0.4 was applied to filter the results. The filtered results from STRING were then imported into Cytoscape software (version 3.9.0) to construct a PPI network. We utilized the CentiScaPe 2.2, a Cytoscape plug-in, to identify key nodes in large networks [32]. ClueGO, another Cytoscape plug-in, was employed to perform GO analysis and visualize the functional genes within a clustered network [33, 34].

### Molecular docking

We downloaded the 3D structures of proteins listed in Table 1 from the Structural Bioinformatics Protein Data Bank (RCSB PDB) database (https://www.rcsb.org/) [35, 36]. Water molecules and excess ligands present in the protein structures were removed using PyMOL software. The chemical structure of laduviglusib was obtained from the PubChem database (https://pubchem.ncbi.nlm.nih.gov/). Molecular docking analysis was conducted to predict the binding sites and interaction forces between proteins and laduviglusib using AutoDock Vina software and SwissDock (http://www.swissdock.ch/) [37–39]. The binding energy and optimal binding conformations were obtained and visualized using PyMOL software. The Protein Ligand Interaction Profiler (PLIP) (https://plip-tool.biotec.tu-dresden.de/plip-web/plip/index) was used to detect non-covalent interactions between macromolecules and laduviglusib, while also providing atom-level insights into the binding characteristics [40]. If necessary, OpenBabel (https://openbabel.org/wiki/Main_Page) was employed to convert file formats.

## Results

### Potential target genes of laduviglusib against each cell-type of HD

**Fig. 1:**
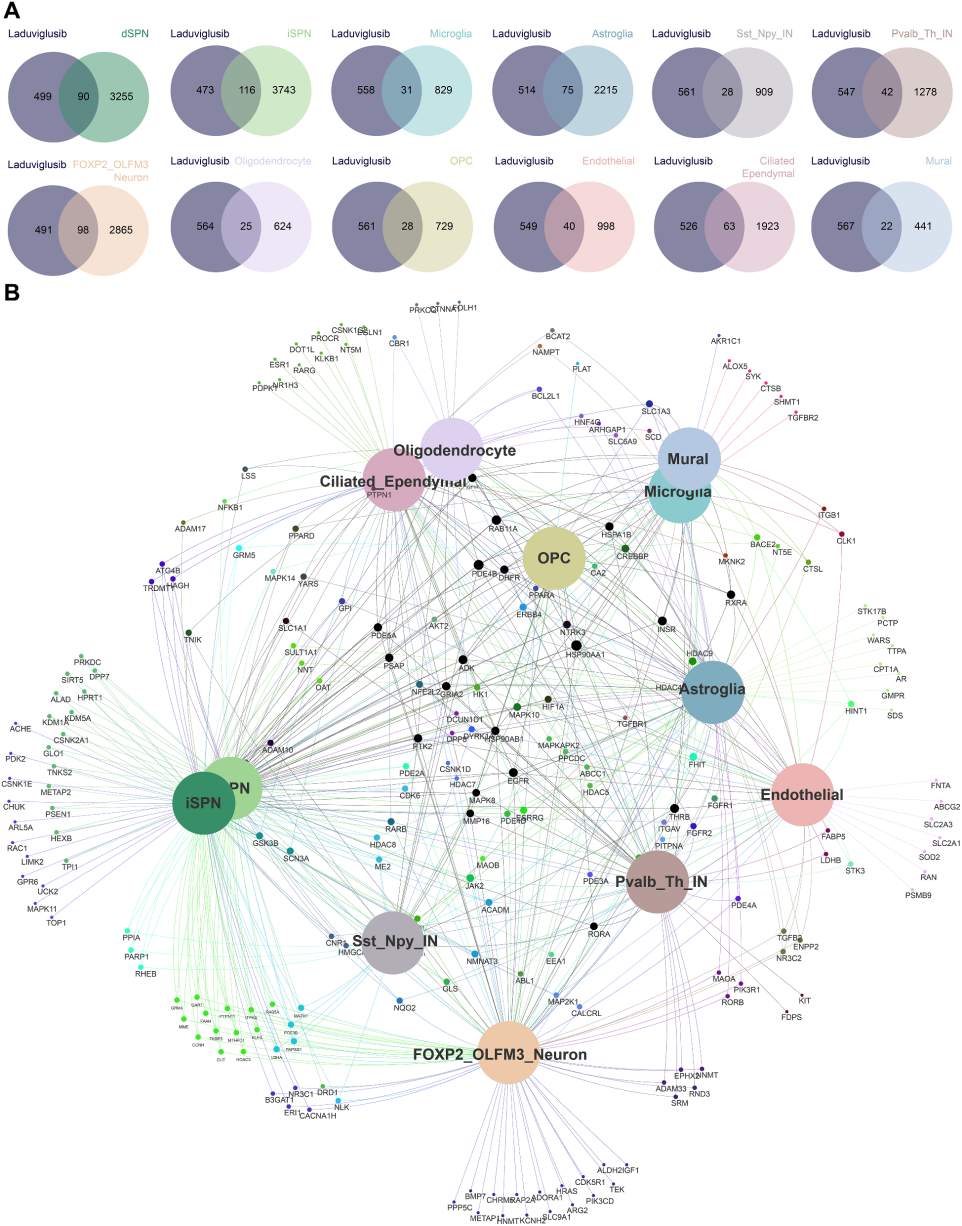
Laduviglusib target different cell-types of HD. (**A** and **B**) The Venn diagrams (**A**) and the Venn network (**B**) visually represents the interconnected genes between different cell-types in HD with laduviglusib

This study focused on the CPu region within the striatum, as these areas are primarily implicated in the manifestation of abnormal movements in HD patients [20, 21]. HD is classified into five grades (0–4) based on various pathological criteria, including the loss of striatal spiny projection neurons (SPNs), the presence of reactive astrocytes, and the degree of microglial activation, reflecting increasing disease severity [1, 3]. To assess the potential target genes of laduviglusib in specific HD cell-types, single-cell sequencing data from post-mortem samples of HD grades 2–4 patients were used. Despite the well-established causative mutation of HD involving the expansion of CAG short tandem repeats in the huntingtin gene, the mutation affects various neuronal subtypes to varying degrees. Differentially expressed genes were selected from distinct cell-types within the CPu region to represent potential target genes for HD patients. The potential target genes of laduviglusib were gathered from multiple databases, with redundancies removed. The identification of the potential target genes of laduviglusib against specific HD cell-types involved the intersection of different cell-types with the potential targets of laduviglusib (Fig. 1) (Supplemental Table S1-S3).

### Potential signaling pathways for laduviglusib in different cell-types of HD

**Fig. 2:**
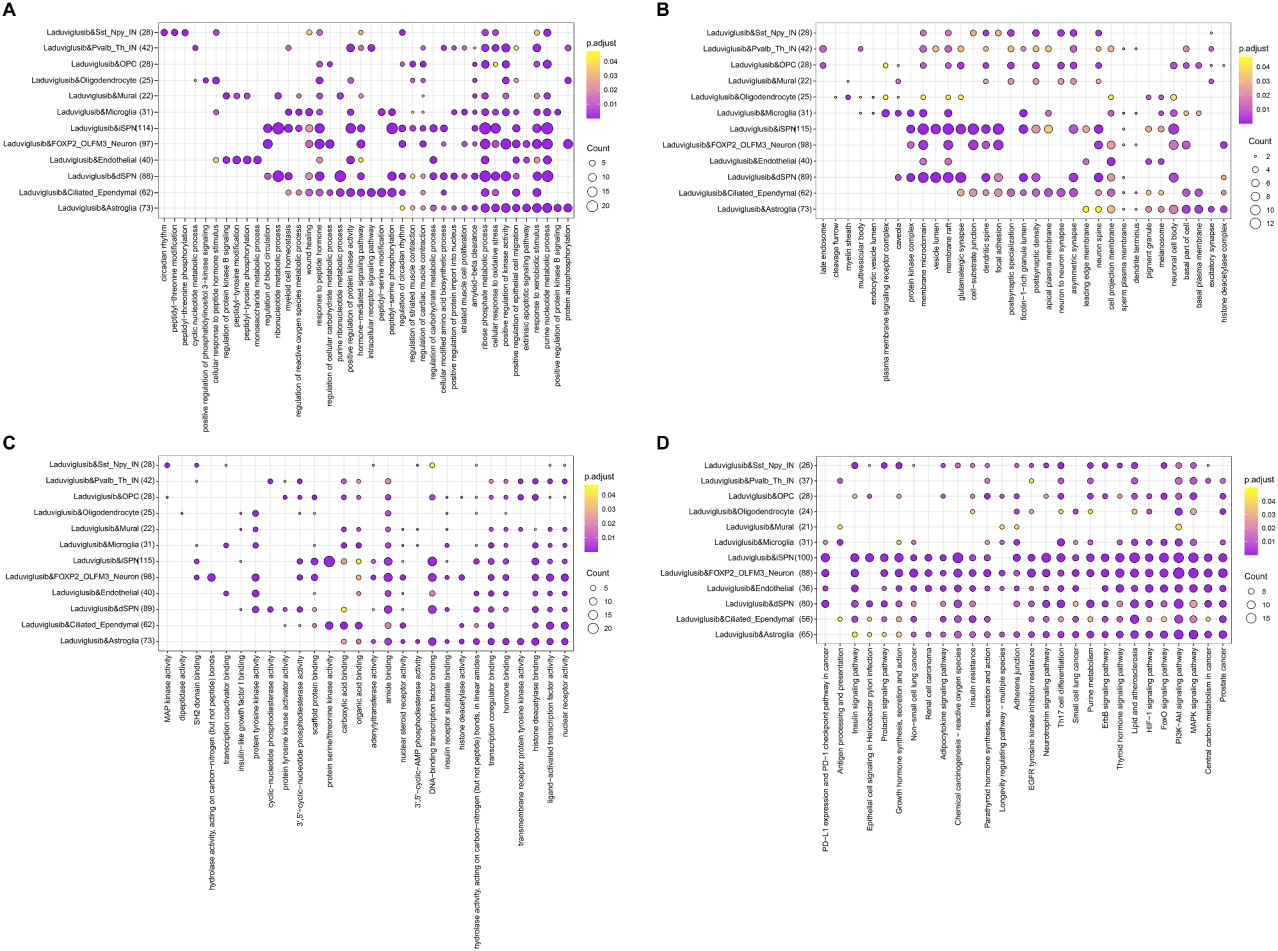
GO and KEGG enrichment analysis of Laduviglusib target genes in different cell-types of HD. Enrichment analysis of laduviglusib targets in different cell-types relevant to HD. The analysis includes (**A**) biological processes, (**B**) cellular components, (**C**) molecular functions, and (**D**) KEGG pathways. Each bubble represents a gene set, with size indicating the number of genes, and color denoting pathway significance

Enrichment analysis revealed the multifaceted impact of laduviglusib on distinct cellular subtypes associated with HD. The highest enrichment scores for Biological Processes (BP), Cellular Components (CC), Molecular Functions (MF), and KEGG pathways related to the target genes of laduviglusib are depicted in Fig. 2 and elaborated upon in Supplemental Table S4 and S5.

As shown in Fig. 2A, in the context of direct pathway striatal projection neurons (dSPNs), laduviglusib significantly modulated biological processes such as purine ribonucleotide metabolism, ribonucleotide metabolism, ribose phosphate metabolism, purine nucleoside metabolism, and amino acid biosynthesis via cellular modification. For indirect pathway striatal projection neurons (iSPNs), the impact of laduviglusib extended to processes such as ribonucleotide metabolism, ribose phosphate metabolism, purine nucleoside metabolism, cellular responses to oxidative stress, and reactions to oxidative stress. In astrocytes, laduviglusib affected processes encompassing protein autophosphorylation, positive regulation of protein kinase B signaling, purine nucleoside metabolism, responses to exogenous stimuli and external apoptotic signaling. Additionally, it exhibited a positive regulatory influence on epithelial cell migration and kinase activity. In microglia, the potential role of laduviglusib was identified in the regulation of reactive oxygen species metabolism, suggesting its contribution to ameliorating HD-related dysregulations.

Figure 2B primarily highlights the therapeutic effects of laduviglusib on Cellular Components in most HD cells, including neuron spine, neuronal cell body, membrane microdomain, and membrane raft.

In Fig. 2C, laduviglusib was found to intervene in Molecular Functions across most HD cell-types, impacting nuclear receptor activity, ligand-activated transcription factor activity, amide binding, et al. Notably, it exhibited different regulatory patterns between dSPNs and iSPNs, with a particular focus on protein serine/threonine kinase activity in iSPNs.

Figure 2D collectively illustrate the most enriched KEGG pathways, providing insights into the probable mechanisms of action of laduviglusib in HD. Notable pathways associated with dSPNs include the Th17 cell differentiation, PD-L1 expression, and the PD-1 checkpoint pathway in cancer, as well as purine metabolism. In contrast, the impact of laduviglusib on iSPNs extends to the Neurotrophin signaling pathway, FoxO signaling pathway, and the Chemical carcinogenesis - reactive oxygen species pathway. In astrocytes, it was found to be implicated in HD through the MAPK signaling pathway, PI3K-Akt signaling pathway, FoxO signaling pathway, Ras signaling pathway, and HIF-1 signaling pathway. For microglia, laduviglusib may play a role in HD via the Th17 cell differentiation and Autophagy. The aforementioned results indicated that a majority of cells are closely associated with HIF-1 signaling pathway, FoxO signaling pathway, PI3K-Akt signaling pathway, MAPK signaling pathway, and Chemical carcinogenesis - reactive oxygen species.

Collectively, these findings underscore the ability of laduviglusib to modulate a spectrum of critical biological processes, including nucleotide metabolism, reactive oxygen metabolism, and oxidative stress responses, thus offering valuable insights into its potential as a therapeutic intervention for HD.

### Ferroptosis-related genes in laduviglusib against each cell-type of HD

**Fig. 3:**
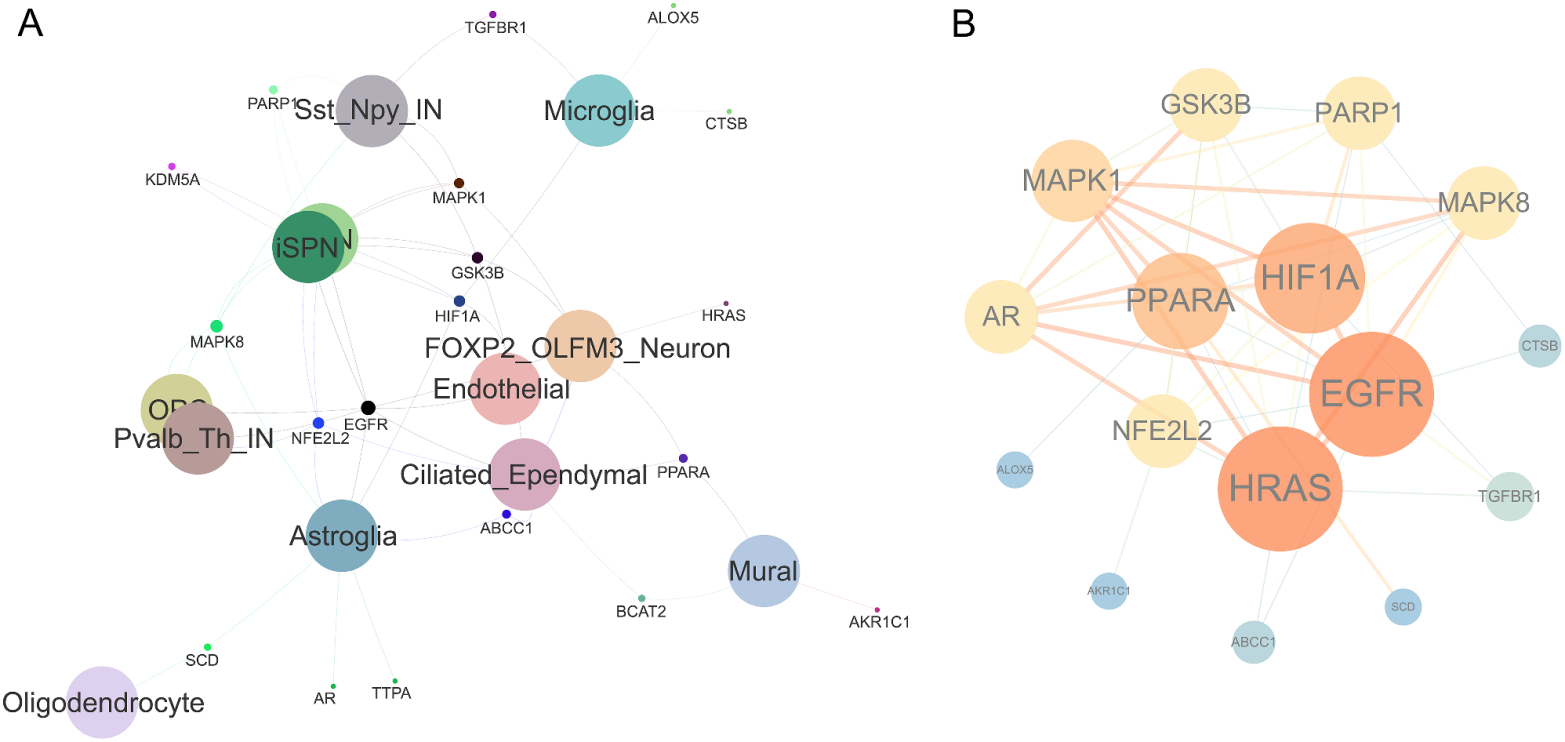
Identification of the potential targets of laduviglusib in treating HD in the context of ferroptosis. (**A**) The Venn network visually represents the interconnected genes shared among HD, laduviglusib, and ferroptosis. (**B**) The analysis of Protein-Protein Interactions (PPI) networks reveals candidate targets of laduviglusib concerning HD treatment within the context of ferroptosis. The node size and edge thickness directly correspond to the degree and betweenness centrality scores, reflecting the extent of connectivity between nodes

Based on the previous enrichment analysis results, there is a compelling rationale to suggest that the protective effects of laduviglusib against HD may be linked to ferroptosis. Consequently, we conducted an intersection analysis among three datasets: Ferroptosis-related genes, laduviglusib target genes, and differentially expressed genes in distinct HD cell-types. The intersection genes are visually represented in a Venn diagram [60], as depicted in Supplementary Fig. S1 (detail listed in Supplemental Table S6). All intersecting data points are interconnected, forming a Venn relationship network diagram (Fig. 3A). Furthermore, utilizing the CentiScape plugin in Cytoscape, we constructed a Protein-Protein Interaction (PPI) network of the targets of laduviglusib in different HD cell-types under the conditions of Closeness (0.03973360452816236), Betweenness (11.374999999999998), and Degree (5.875) (Fig. 3B). This network analysis provides insights into the potential mechanisms underlying the impact of laduviglusib on distinct cell-types in HD.

### Poteintial signaling pathways related to ferroptosis induced by laduviglusib in distinct cell-types of HD

**Fig. 4:**
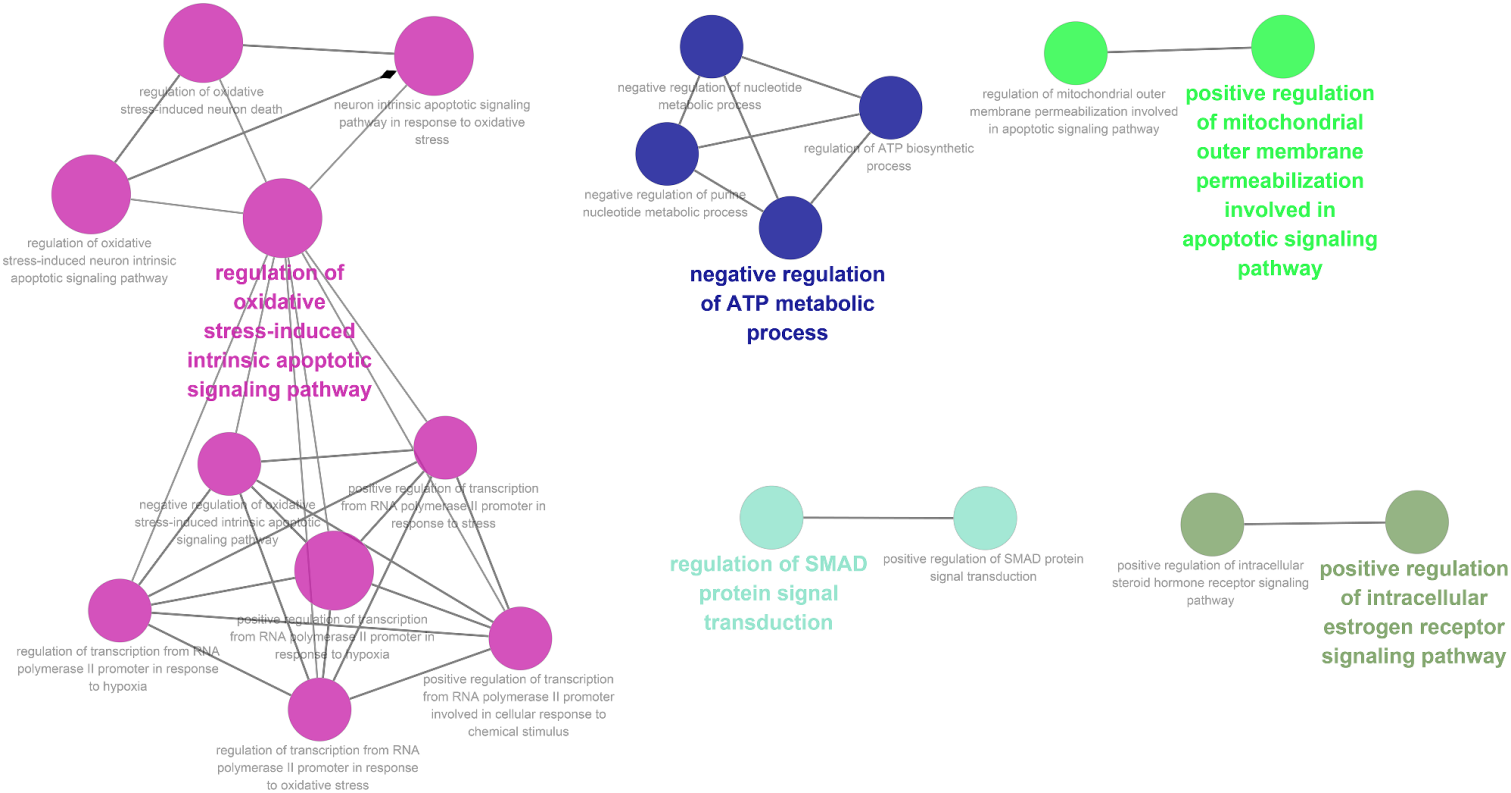
GO enrichment analysis. GO enrichment analysis of Laduviglusib Target genes in different cell-types of HD through Ferroptosis through the Cytoscape plugin ClueGO

To preliminarily explore the potential functions of the target genes of laduviglusib in different cell-types related to ferroptosis, we conducted enrichment analysis using the Cytoscape plugin ClueGO. The enrichment results indicated that these genes are involved in functions such as the regulation of oxidative stress-induced intrinsic apoptotic signaling pathway, negative regulation of ATP metabolic processes, and positive regulation of mitochondrial outer membrane permeabilization in the apoptotic signaling pathway (Fig. 4).

**Fig. 5:**
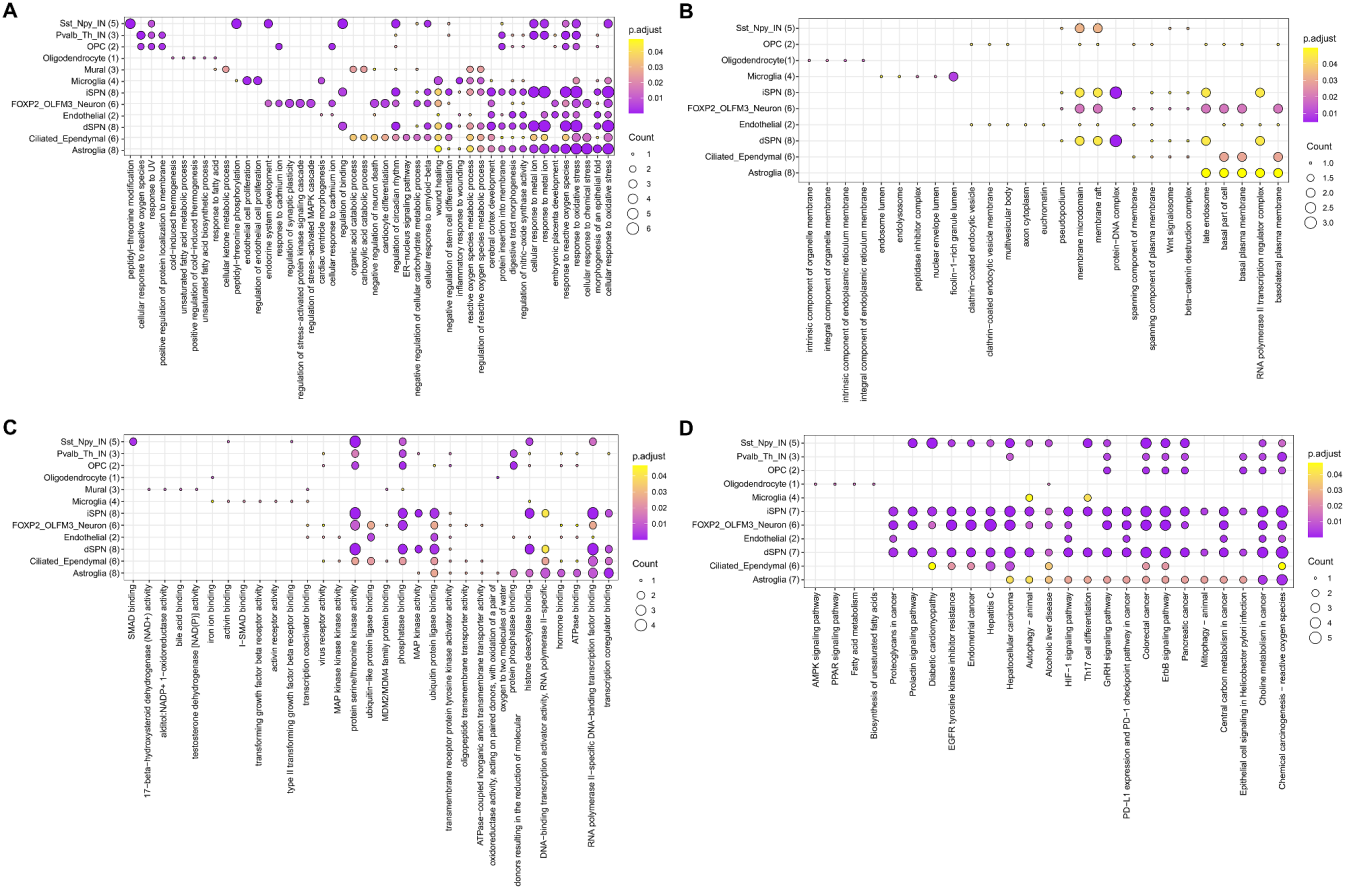
GO and KEGG enrichment Analysis of laduviglusib target genes in HD different cell-types through ferroptosis. Enrichment analysis of laduviglusib targets in different cell-types relevant to HD through ferroptosis. The analysis includes (**A**) biological processes, (**B**) cellular components, (**C**) molecular functions, and (**D**) KEGG pathways. Each bubble represents a gene set, with size indicating the number of genes and color denoting pathway significance

To elucidate the potential signaling pathways targeted by laduviglusib in the context of HD, we conducted GO and KEGG enrichment analyses on the genes targeted by laduviglusib that are associated with ferroptosis across different cell-types (Supplemental Table S7 and S8). The results revealed that the influence of laduviglusib on the ferroptosis-related biological processes in most HD cells primarily centered around response to reactive oxygen species, response to oxidative stress, and cellular response to oxidative stress (Fig. 5A).

Moreover, in both dSPNs and iSPNs, specific cellular components such as protein-DNA complex and molecular functions including protein serine/threonine kinase activity, phosphatase binding, ubiquitin protein ligase binding, and RNA polymerase II-specific DNA-binding transcription factor binding, emerged as particularly critical (Fig. 5B, C).

Furthermore, KEGG enrichment analysis indicated that laduviglusib may exert its protective effects on different cell-types in HD, including astroglia, dSPNs and iSPNs, by interacting with the Chemical carcinogenesis - reactive oxygen species signaling pathway, which is related to ferroptosis. In microglia, laduviglusib may exert its protective effects on HD through Th17 cell differentiation and Autophagy (Fig. 5D).

These findings collectively provide valuable insights into the potential signaling pathways through which laduviglusib operates in the context of HD, particularly in relation to ferroptosis, across distinct cell-types.

### Molecular docking

**Fig. 6:**
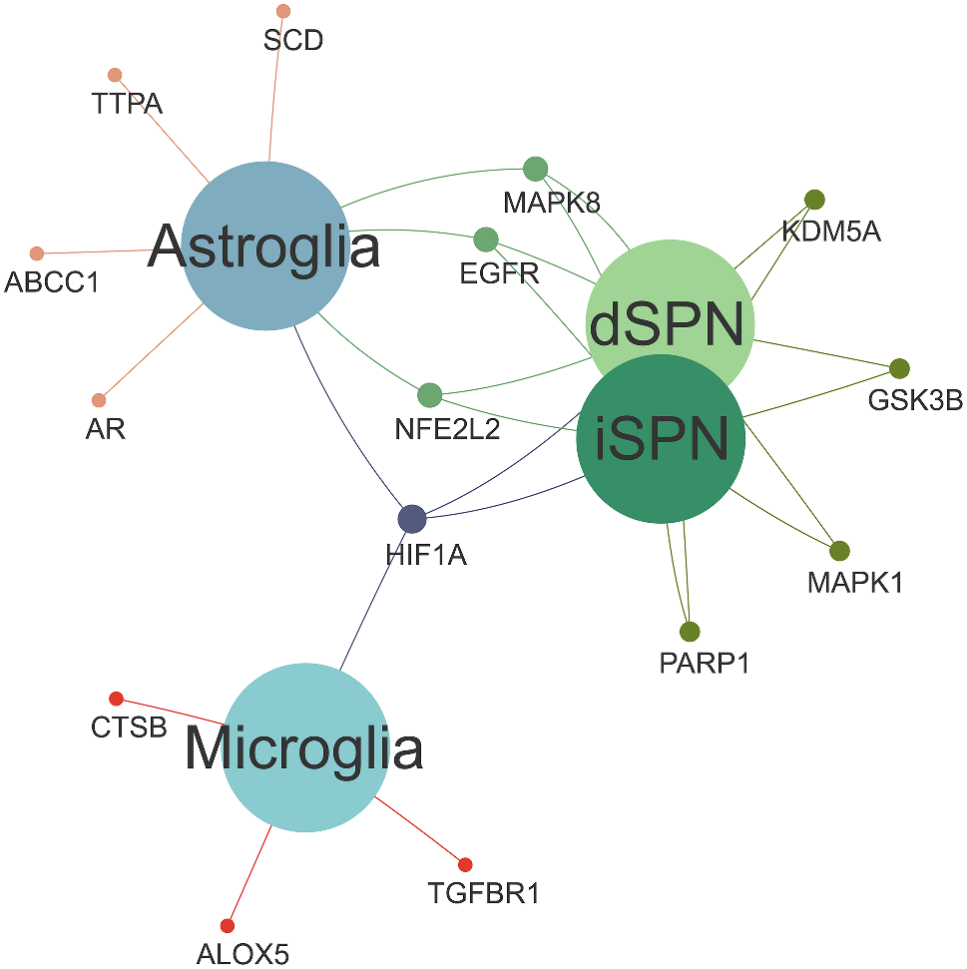
Venn network The Venn network visually represents the interconnected genes shared among laduviglusib, ferroptosis, and HD different cell-type, including astroglia, dSPNs, iSPNs, microglia

**Fig. 7:**
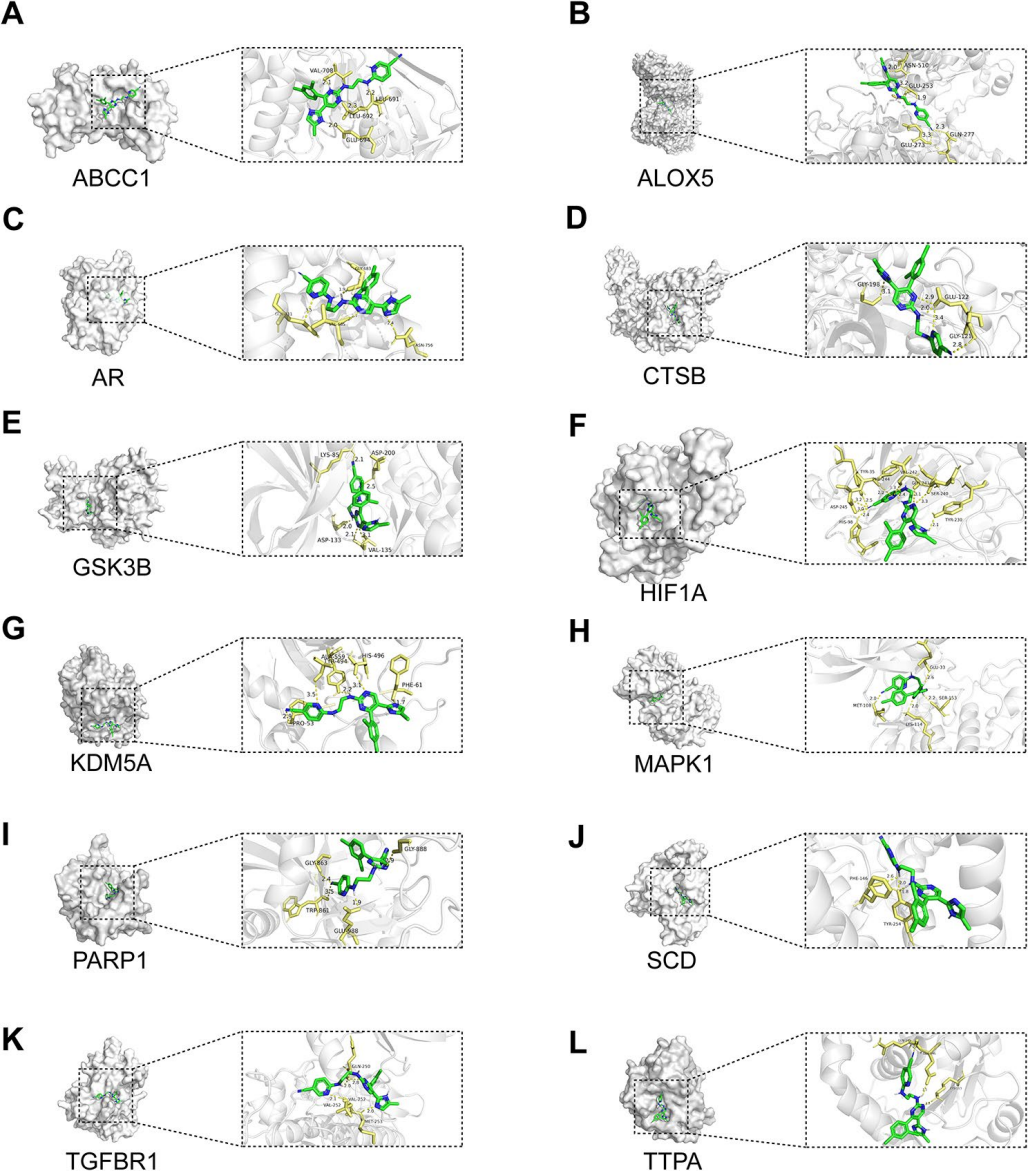
Schematic diagram of molecular docking for laduviglusib. (**A**–**E**) 3D diagrams represent the molecular binding of laduviglusib with ABCC1 (**A**), ALOX5 (**B**), AR (**C**), CTSB (**D**), GSK3B (**E**), HIF1A (**F**), KDM5A (**G**), MAPK1 (**H**), PARP1 (**I**), SCD (**J**), TGFBR1 (**K**), TTPA (**L**), respectively

The progression of Huntington’s Disease (HD) predominantly impacts spiny projection neurons (SPNs) in the striatum, with Drd2-expressing iSPNs being particularly susceptible compared to Drd1-expressing dSPNs [1, 41]. This complex pathogenesis involves concurrent neuroinflammatory processes, characterized by microglial activation preceding clinical symptoms and reactive astrogliosis occurring later in the disease course [41]. To assess the potential molecular interactions between laduviglusib and genes associated with ferroptosis processes in dSPNs, iSPNs, astrocytes, and microglia, molecular docking studies were conducted (Figs. 6 and 7). The detail binding information of laduviglusib for each target protein were listed in Table 1 and Supplemental Table S9-11. The binding energy of docking of all proteins with laduviglusib were less than − 6.96 kcal/mol, indicating a tight binding between the proteins and laduviglusib. The top five proteins were PARP1 (associated with dSPNs and iSPNs), SCD (associated with astrocyte), AR (associated with astrocyte), ALOX5 (associated with microglia), HIF1A (associated with dSPNs, iSPNs, astrocyte, microglia). These data suggest that laduviglusib exerts its anti-HD effects by influencing distinct cellular functions, with multiple ferroptosis-related signaling pathways potentially mediating this effect.

**Table 1:** Molecular docking binding energy results

Protein	PDB ID	Binding Energy (kcal/mol)
ABCC1	2cbz	-7.7
ALOX5	3o8y	-7.85
AR	4hlw	-7.97
CTSB	5mbm	-7.24
GSK3B	6tcu	-7.23
HIF1A	1h2m	-7.83
KDM5A	5e6h	-7.61
MAPK1	6g54	-7.61
PARP1	5ds3	-8.16
SCD	4zyo	-8.06
TGFBR1	6b8y	-7.68
TTPA	5mue	-6.96

## Discussion

In this study, we employed single-cell sequencing data obtained from post-mortem samples of HD patients in stages 2–4 to identify gene expressions across different cell-types in the CPu. Through the integration of these gene profiles with the putative target genes of laduviglusib, our research has unveiled the regulatory role of laduviglusib across various cell types within the HD striatum, with a particular emphasis on dSPNs, iSPNs, astrocytes, and microglia. This regulatory influence extends to processes linked to ribose metabolism and oxidative stress. We highlight that the effects of laduviglusib in neuronal dendritic spines and somas of the iSPNs and dSPNs may reverse the neuropathological changes related to movement disorders observed in HD. Molecular docking results indicated that laduviglusib binding well with PARP1 (associated with dSPNs and iSPNs), SCD (associated with astrocyte), AR (associated with astrocyte), ALOX5 (associated with microglia), HIF1A (associated with dSPNs, iSPNs, astrocyte, microglia). In addition, the KEGG and molecular docking results suggest that laduviglusib may enhance mitochondrial function and protect against neuronal loss by targeting ferroptosis-related signaling pathways, particularly mediated by ALOX5 in microglia according to the KEGG analysis. We determined the protective role of laduviglusib in cell-type-specific in HD striatum, which provides insights into HD therapy.

This research underscores the potential therapeutic value of laduviglusib in the context of neurodegenerative disorders. Laduviglusib serves as a highly selective inhibitor of GSK3 while simultaneously activating the Wnt/β-catenin signaling pathway, a crucial component for cell viability preservation. Furthermore, it has shown promise in enhancing insulin-mediated glucose transport, promoting pancreatic β-cell proliferation, and ensuring their survival [7, 8]. In the context of Alzheimer’s disease, laduviglusib has exhibited the ability to reduce tau phosphorylation in glutamatergic neurons, suggesting a potential role in counteracting tau accumulation, a hallmark of Alzheimer’s disease [13]. Recent in vitro studies have also highlighted the capacity of laduviglusib to salvage microtubule protein aggregation within ankyrin 3-inhibited cells by pharmacologically inhibiting CRMP2 activity [14]. Most notably, in HD, laduviglusib has demonstrated its potential in inhibiting the proteolysis of calpastatin protein bodies, impeding Drp1 recruitment to mitochondria, and reducing mitochondrial fragmentation [24]. This mechanism provides a possible route for inhibiting mitochondrial damage, enhancing neuronal viability in HD mouse models and patients, and safeguarding against HD-related neuropathological changes.

As is widely recognized, HD primarily stems from the aggregation of toxic large-molecule proteins formed due to mutations in the Htt gene. The severity of these aggregates varies based on the cell-types they accumulate in and their specific locations [6, 42]. In neuronal cytoplasm, these accumulations have the potential to disrupt systems responsible for managing abnormal proteins, including proteasomes, chaperone proteins, and autophagic proteins. In the neuronal nucleus, these toxic large molecules can disrupt the transcription of antioxidant genes. Moreover, in glial cells, the expression of mutant Htt (mHtt) stimulates immune cells to secrete pro-inflammatory cytokines, further exacerbating mitochondrial dysfunction and disrupting the redox state of neurons [43]. These observations emphasize the significance of comprehending how different cell-types contribute to HD pathology in the context of laduviglusib treatment. Our research demonstrates that laduviglusib influences ribose metabolism and elicits responses to oxidative stress in both dSPNs, iSPNs, astrocytes, microglia, and other cell-types. Furthermore, we noted that it regulates potential ROS metabolism in microglia and iSPNs. Importantly, it’s worth highlighting that laduviglusib exhibits distinct regulatory patterns between dSPNs and iSPNs, with a particular emphasis on regulating serine/threonine kinases in iSPNs. This differential regulatory pattern extends to the level of signaling pathways. For instance, potential pathways influenced by laduviglusib in dSPNs encompass the HIF-1 signaling pathway, neurotrophin signaling pathway, and PI3K-Akt signaling pathway. In contrast, its effects on iSPNs are associated with neurotrophin signaling, FoxO signaling, and the chemical carcinogenesis-ROS pathway. These unique regulatory patterns underscore the notion that different cell-types play distinct roles in the development of motor dysfunction in HD. SPNs in HD patients exhibit varying susceptibilities, with iSPNs being more vulnerable compared to dSPNs and other cell-types [1, 44]. Moreover, we found that laduviglusib affects dendritic spines and somas of iSPNs and dSPNs. This indicates that the mechanism of laduviglusib is closely related to abnormal dendritic spine changes and neuronal death. Compelling evidence suggests that the deficiency of the mHtt in HD synapses leads to synaptic changes, including dendritic spine enlargement, but these changes result in decoupled synaptic function, ultimately leading to the death of HD neurons [45]. This is pivotal in the ultimate development of HD symptoms. KEGG enrichment analysis unveiled shared signaling pathways, with the PI3K-Akt signaling pathway notably among them. This is likely due to laduviglusib being a highly selective GSK3 inhibitor and Wnt/β-catenin signaling activator. Recent studies have shown that laduviglusib can inhibit the activation of Akt, PI3K, nuclear factor-κB induced by IL-1β and TNF-α in cells modeling Graves’ orbitopathy [46]. This reverses the inhibitory effects of Wnt and β-catenin in the fat generation process, reduces the production of pro-inflammatory cytokines, and inhibits fibroblast differentiation into fat cells [46]. However, Di et al. recently discovered that laduviglusib can inhibit the degradation of the calpastatin protein, suppress Drp1 recruitment to mitochondria, reduce mitochondrial fragmentation, and thus inhibit mitochondrial damage [24]. This leads to improved cell viability in HD mice and patients, suggesting that laduviglusib may enhance mitochondrial function in HD neurons by targeting the CAST/calpain/Drp1 pathway, rather than GSK3, thus providing protection against HD. All these findings underscore the notion that laduviglusib likely exerts its protective effects in HD by modulating signaling pathways in different cell-types, ultimately enhancing mitochondrial function and ameliorating HD-associated functional impairments.

In HD, the neuroprotective effects of laduviglusib stem from its modulation of the CAST-calpain-Drp1 pathway [24]. Numerous studies have highlighted the pivotal roles of calpain and Drp1 in ferroptosis. The modulation of ferroptosis by calpain is linked to its potential for preventing the onset of Alzheimer’s disease-like phenotypes and its involvement in the pathogenesis of systemic sclerosis [25, 27]. Consistently, enhanced Drp1 activity is reported to facilitate ferroptosis by regulating mitochondrial fission [28–30]. These findings collectively suggest that the activation of calpain/Drp1 may lead to mitochondrial and lysosomal damage, thereby promoting or exacerbating ferroptosis. Furthermore, prior studies have hinted at the potential role of laduviglusib through mitochondrial oxidative stress-related pathways. It has been observed to significantly oxidative damage, including the elevation of ROS levels, mitochondrial membrane potential disruption, excessive lipid peroxidation, and decreased SOD activity [15]. These suggest that laduviglusib could be involved in mitigating oxidative-related functional impairments. In line with these findings, our enrichment analysis has uncovered that laduviglusib mediates oxidative stress responses in dSPNs, iSPNs, astrocytes, and microglia within HD. Oxidative stress and mitochondrial function are closely associated with iron-dependent cell death processes, notably ferroptosis, which is a non-apoptotic form of cell death linked to lethal iron-dependent lipid peroxidation [16]. Studies have illustrated that under conditions of oxidative stress, neurons tend to undergo ferroptosis, emphasizing the role of iron in oxidative stress-induced neurodegeneration [47]. Previous research has established the pivotal role of ferroptosis in the pathogenesis of HD. Iron accumulation has been identified in both HD mouse models and patients [17, 18]. The regulation of iron, especially within mitochondria, is crucial for metabolic processes. For instance, mitochondrial iron accumulation has been detected in the brain tissues of HD patients and HD mice, leading to mitochondrial dysfunction and increased lipid peroxidation [19]. Accumulation of lipid peroxidation in the CPu has been closely associated with abnormal movements in HD patients [20, 21]. This aligns with prior findings by Di et al. [24], raising reasonable suspicion that laduviglusib might regulate ferroptosis-related pathways to enhance mitochondrial function and counteract HD-related neuropathological changes.

Pharmacological agents targeting ferroptosis pathways have shown immense potential in the clinical treatment of HD [22]. Earlier studies have demonstrated that ferrostatin-1, which selectively inhibit iron oxidation, can rescue neurons from death in HD cell models [23]. Treating HD mice with iron chelator deferiprone restores the normal mitochondrial iron content in the striatum and cortex, leading to improved mitochondrial membrane potential, oxygen consumption, reduced lipid peroxidation, and increased ATP and GSH levels [19]. In our study, we discovered that laduviglusib primarily impacts ferroptosis-related processes in most HD cells, including dSPNs, iSPNs, astrocytes, microglia. This impact centers around responses to reactive oxygen species, oxidative stress, and cellular reactions to oxidative stress. KEGG enrichment analysis predicts that laduviglusib may interact with active oxygen signaling pathways related to ferroptosis to protect astrocytes and both dSPNs and iSPNs in HD. Molecular docking results found that laduviglusib binding well with PARP1 (associated with dSPNs and iSPNs), SCD (associated with astrocyte), AR (associated with astrocyte), ALOX5 (associated with microglia), HIF1A (associated with dSPNs, iSPNs, astrocyte, microglia). Numerous evidence also indicated that PARP1, SCD, AR, ALOX5, HIF1A are critical involved in HD or neurodegenerative diseases [48–54]. These results indicated that these proteins may play crucial role in ferroptosis-related pathway in HD interact with laduviglusib. For instance, we found several genes, particularly HIF1A, exhibit interactions with multiple cell types. Notably, our findings revealed that HIF-1α expression is perturbed in neurons, microglia, and astrocytes. HIF-1α is a pivotal regulator of ROS in neurons, where it upregulates Drp1 expression, resulting in mitochondrial fragmentation and elevated ROS production [55]. Following stress or trauma, HIF-1α activation in microglia leads to increased ROS production and subsequent inflammation, potentially causing cellular damage [56]. Moreover, mutation of the Surf1 gene activates a mitochondrial ROS-HIF-1α signaling cascade that stimulates astrocytic glycolysis, mitigating the hypersensitivity of dopaminergic neurons to trauma-induced degeneration [57]. Conversely, acute MeHg exposure decreases HIF-1α in astrocytes via PHD and the UPS, suppressing ROS production and exacerbating neurotoxicity [58]. The activation of HIF-1α in various neural cells is critical for the generation of reactive oxygen species, underscoring its pivotal role in the pathogenesis of Huntington’s disease. Notably, we found that the effect of laduviglusib on HD microglia in counteracting ferroptosis is mediated by HIF1A and ALOX5 through Th17 cell differentiation and autophagy signaling pathways, respectively. The latter discovery parallels the findings of Song et al., who uncovered that HTT fragment expression induces ferroptosis under ROS-induced stress conditions via ALOX5 mediation [25]. Recent research also highlights microglia cells as key players in cellular iron oxidation cascades, with their unique neurotoxic status contributing to neurodegenerative diseases [59]. Removing microglia cells in a three-cell culture system reduces lipid peroxidation in neurons and significantly delays neuronal death under iron dysregulation conditions [59]. This evidence indicates that ferroptosis-related mechanisms play a crucial role in the pathophysiological development of HD in various cell-types. Combined with our current research, it’s reasonable to suspect that laduviglusib may exert its therapeutic properties in HD by targeting different cell-type-specific ferroptosis-related signaling pathways. These findings offer valuable insights into the potential signaling pathways of laduviglusib within the context of HD, especially those associated with iron oxidation in various cell-types.

It is regrettable that our current study has not yet encompassed the experimental validation of several key genes. Due to the absence of experimental corroboration, the conclusions of this research are subject to caveats, particularly with regard to the predicted interactions of laduviglusib with multiple proteins, which require further in vitro or in vivo laboratory investigation. This gap in our research will be addressed through the design of supplementary experimental validations in future studies.

## Conclusion

In conclusion, this study enhances our comprehension of the potential signaling pathways affected by laduviglusib in the context of HD, shedding light on its multifaceted impact across diverse cell types, particularly with regard to ferroptosis-related signaling pathways. These findings represent a promising starting point for future investigations, encompassing animal and patient validation studies, as well as an in-depth examination of the distinctive regulation of ferroptosis by laduviglusib in dSPNs, iSPNs, and microglia cells. These forthcoming endeavors hold the potential to greatly enhance our knowledge of the applications of laduviglusib in the intricate landscape of neurodegenerative diseases.

### Electronic supplementary material

Below is the link to the electronic supplementary material.

Supplementary Material 1

Supplementary Material 2

Supplementary Material 3

Supplementary Material 4

Supplementary Material 5

Supplementary Material 6

Supplementary Material 7

Supplementary Material 8

Supplementary Material 9

Supplementary Material 10

Supplementary Material 11

Supplementary Material 12

## Data Availability

The original contributions presented in the study are included in the article/ Supplementary Material. Further inquiries can be directed to the corresponding authors.

## Abbreviations

HD: Huntington’s disease
KEGG: The Kyoto Encyclopedia of Genes and Genomes
GO: Gene Ontology
dSPNs: Direct pathway striatal projection neurons
iSPNs: Indirect pathway striatal projection neurons
HTT: Huntingtin
SPNs: Striatal spiny projection neurons
ROS: Reactive oxygen species
SOD: Superoxide dismutase
CPu: Caudate nucleus and putamen
AD: Alzheimer’s disease
SSc: Systemic sclerosis
HCC: Hepatocellular carcinoma
snRNA-seq: Single-nucleus RNA sequencing
FDR: False discovery rate
SMILES: Simplified Molecular Input Line Entry System
BP: Biological processes
CC: Cellular components
MF: Molecular functions
PPI: Protein-Protein Interaction
RCSB PDB: Structural Bioinformatics Protein Data Bank
PLIP: Protein Ligand Interaction Profiler
mHtt: Mutant Htt
