# Supplementary material for: Uncovering the ferroptosis related mechanism of laduviglusib in the cell-type-specific targets of the striatum in Huntington’s disease
Source: BMC Genomics. 2024 Jun 25;25:633. doi: 10.1186/s12864-024-10534-5 (PMC11197352; doi:10.1186/s12864-024-10534-5)
Supplement: Supplementary file 1 — Supplementary Material 1 [file 12864_2024_10534_MOESM1_ESM.docx]

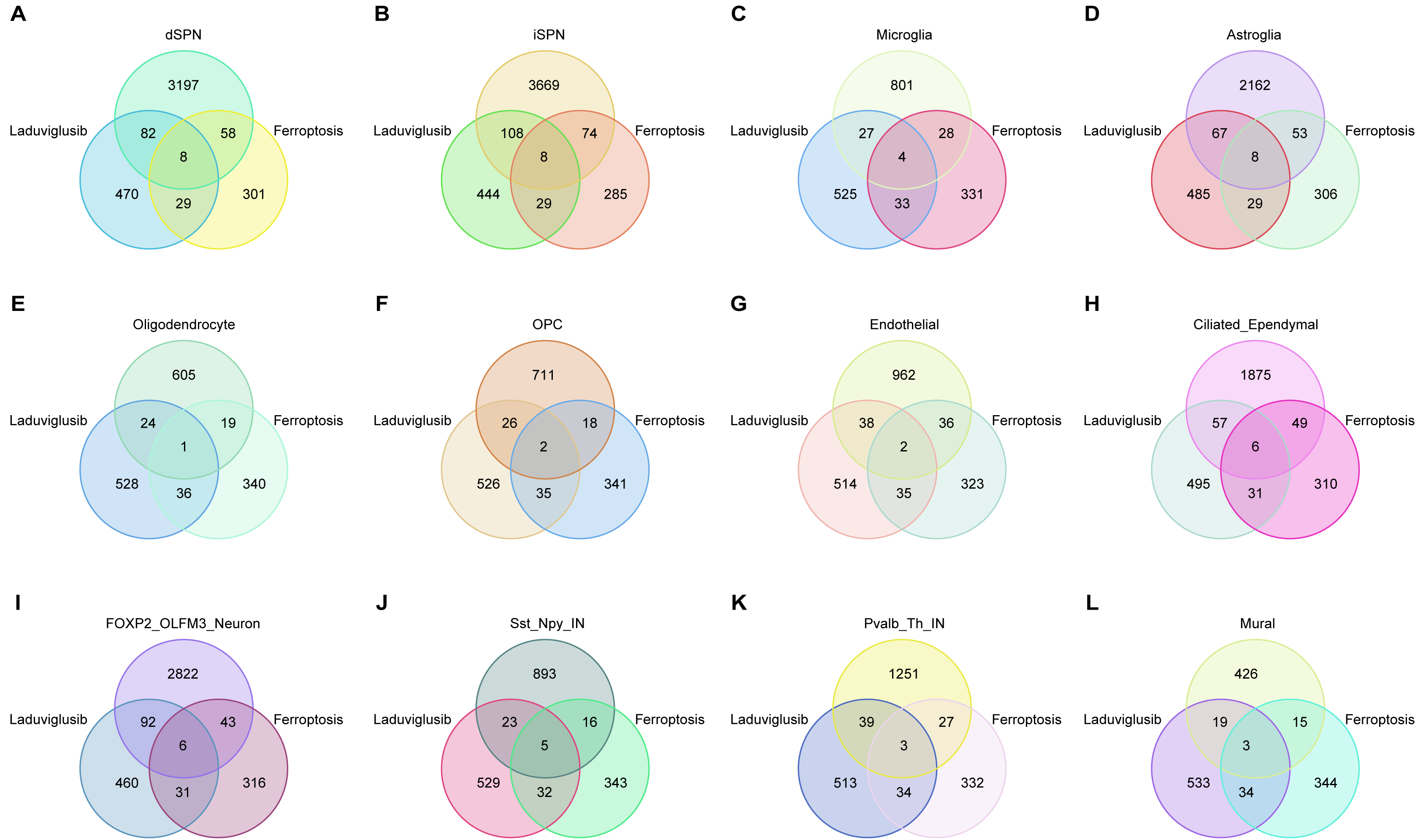


**Figure S1. Intersections of all cell-types associated with ferroptosis**

The Venn diagram illustrates the target genes that intersect among laduviglusib, ferroptosis, and differentially expressed genes in distinct cell-types associated with HD.
